# A Temporal Candidemia Cluster in a Jordanian Pediatric Oncology Unit: Clinical Characterization, Putative Environmental Sources, and Multidisciplinary Infection Control Responses—A Single-Center Pilot Study

**DOI:** 10.3390/jof12080556

**Published:** 2026-07-27

**Authors:** Rawan N. Budair, Dana Kanaan, Tala Al Shalakhti, Hiba Emadeldeen, Joud Jarrah, Rawad Rihani

**Affiliations:** Pediatric Oncology Department and Infection Control Program, King Hussein Cancer Center, Amman 11941, Jordan; danakanaan995@gmail.com (D.K.); ta.16852@khcc.jo (T.A.S.); ha.17051@khcc.jo (H.E.); jj.11090@khcc.jo (J.J.); rrihani@khcc.jo (R.R.)

**Keywords:** *Candida tropicalis*, candidemia, temporal cluster, pediatric oncology, antifungal resistance, infection prevention and control, low- and middle-income countries, Jordan

## Abstract

**Background:** Candidemia carries case-fatality rates exceeding 30% in pediatric oncology cohorts from low- and middle-income countries. We describe a temporal cluster of culture-confirmed candidemia cases at King Hussein Cancer Center (KHCC), Amman, Jordan, coinciding with a novel environmental event and the multidisciplinary infection prevention and control (IPC) response undertaken. **Methods:** This retrospective cohort study describes seven pediatric inpatients with culture-confirmed candidemia identified between June 1 and September 30, 2025, at a 330-bed tertiary oncology center against a background rate of one confirmed case in the preceding six months. An additional five patients with clinically suspected invasive fungal infection (IFI) but negative blood cultures are described separately. Species identification was performed using the BioFire FilmArray Blood Culture Identification (BCID) panel (multiplex PCR); MALDI-TOF MS was additionally available; antifungal susceptibility was determined using the Sensititre system (Thermo Fisher Scientific, UK). **Results:** The seven confirmed cases comprised six males and one female (median age: 12 years; range: 1–17 years). Underlying diagnoses were predominantly leukemia (71%). All patients had a central venous catheter (CVC) in situ and recent broad-spectrum antibiotic exposure. *Candida tropicalis* was the predominant species (57%), with azole resistance identified in 42% of tested isolates. A plumbing infrastructure failure with an associated water leak was identified in late August 2025 during the cluster investigation; structural repair was completed in September 2025. The last confirmed case was recorded on 23 September 2025. All patients received guideline-concordant echinocandin- or amphotericin-based therapy. Candidemia-attributable mortality was 14% (1/7). **Conclusions:** A temporal cluster of seven culture-confirmed candidemia cases—representing a 7-fold increase above background incidence—was identified at a Jordanian pediatric oncology center. The cluster coincided with a documented environmental water leak. Individual single-room isolation, geographic ward sectioning, contact precautions, hand hygiene monitoring, expanded antifungal prophylaxis with real-time protocol adjustment, and environmental remediation were all implemented and documented with defined operational criteria. These data demonstrate that a structured, criteria-driven IPC bundle is operationally feasible at a resource-limited tertiary oncology center.

## 1. Introduction

Candidemia is the most common invasive fungal infection in pediatric oncology wards, with consequences that are disproportionately severe in neutropenic, CVC-dependent patients receiving broad-spectrum antibiotics. Globally, an estimated 1.4 million people develop *Candida* bloodstream infection or invasive candidiasis annually, with a case-fatality rate exceeding 60% [1]. In the largest multinational pediatric candidemia dataset published to date, children admitted to hematology–oncology wards had the highest reported rates of non-*albicans Candida* species of any pediatric ward type, while *C. albicans* and *C. parapsilosis* remained the most frequently isolated species overall [2]. Multiple host factors independently increase risk in this population, including prolonged neutropenia, chemotherapy-related mucosal barrier disruption, CVC use, broad-spectrum antibiotic pressure, and corticosteroid administration [3,4]. Disease burden and outcomes are markedly worse in low- and middle-income country (LMIC) settings, where non-culture diagnostics (β-D-glucan, PCR) are largely unavailable, antifungal formularies are often limited to fluconazole and amphotericin B deoxycholate, and infection control infrastructure is under-resourced—each independently worsening candidemia outcomes [5].

The epidemiology of candidemia in pediatric oncology units across the Middle East and the broader LMIC region is shaped by the predominance of non-*albicans Candida* species—particularly *C. tropicalis*—and rising azole resistance rates that approach 20–50% in some surveillance cohorts [6,7].

Beyond its epidemiological prevalence, *C. tropicalis* exhibits specific virulence attributes that distinguish it from *C. albicans* in the neutropenic host. Superior biofilm formation on vascular catheter surfaces facilitates persistent bloodstream seeding; visceral tropism drives hepatosplenic candidiasis at rates disproportionate to other species; and secreted protease and phospholipase activity promotes tissue invasion [8]. Azole resistance in *C. tropicalis* is predominantly mediated by *ERG11* point mutations—notably Y132F and A114V—and *UPC2*-mediated upregulation of sterol biosynthesis genes, conferring cross-resistance between fluconazole and voriconazole and rendering standard azole prophylaxis ineffective against resistant strains [9]. Echinocandin resistance, driven by *FKS1* and *FKS2* gain-of-function mutations, has been reported in *C. glabrata* and, less commonly, in *C. tropicalis*, representing a serious threat to the last reliable empirical prophylaxis class [10]. Collectively, these mechanisms underscore the imperative for local antifungal resistance surveillance and stewardship-guided prophylaxis selection, rather than empirical azole use in settings where azole resistance prevalence is known to be high [9].

Temporal clusters of candidemia in hematology–oncology units—defined as a higher-than-expected number of cases within a defined period, location, and patient population—have been attributed to environmental sources including hospital water systems, construction activity, and failures in air handling [10,11]. Environmental water sources are of particular relevance because hospital plumbing biofilms can simultaneously harbor multiple *Candida* species, producing mixed-species clusters without clonal transmission [12]. Early recognition of such clusters depends on prospective microbiology surveillance and a clearly defined case definition that separates culture-confirmed from clinically suspected diseases.

This paper describes the clinical and microbiological characteristics of culture-confirmed cases, the possible environmental context, and the multidisciplinary response of the infection control team to a temporary outbreak of candidemia in a tertiary cancer center in Jordan, which occurred between June and September 2025. To our knowledge, this work represents one of the few published studies on candidemia outbreaks in pediatric oncology units in Jordan, contributing to the limited existing literature on candidemia outbreaks in the Levant region.

## 2. Methods

### 2.1. Study Design and Setting

This was a retrospective cohort study conducted at KHCC, Amman, Jordan. The cluster observation period spanned 1 June to 30 September 2025. A documented background rate of 1 culture-confirmed candidemia case in the preceding 6 months (December 2024 to May 2025) served as the epidemiological baseline against which the cluster was defined, consistent with WHO criteria for a temporal cluster [13].

### 2.2. Case Definitions

Culture-confirmed candidemia (primary analysis): Isolation of a *Candida* species from at least one blood culture drawn from a peripheral vein or CVC in a patient with compatible clinical signs. These cases constitute the primary analytical cohort.

Clinically suspected IFI (secondary descriptive group): Patients with host risk factors consistent with modified ECMM/ISHAM criteria for probable IFI and compatible clinical or radiologic findings, but with negative blood cultures throughout. These patients are described separately and are explicitly excluded from all candidemia-specific analyses. The term “candidemia” is not applied to this group.

### 2.3. Inclusion Criteria

Patients aged 18 years or younger, of either sex, admitted to the pediatric oncology unit at KHCC, who had culture-confirmed candidemia (primary analysis) or clinically suspected candidemia consistent with probable invasive fungal infection (secondary descriptive group), within the study timeframe.

### 2.4. Exclusion Criteria

Patients with sepsis or bloodstream infection not attributable to a *Candida* species were excluded.

### 2.5. Data Collection

Data were extracted from electronic medical records. Variables included patient demographics, underlying malignancy and disease status, clinical risk factors, microbiological results, antifungal therapy regimens, and 30-day outcomes.

### 2.6. Microbiological Methods

Blood cultures were incubated using the VersaTREK automated blood culture system (Thermo Fisher Scientific, UK). Species identification from positive blood culture bottles was performed using the BioFire FilmArray Blood Culture Identification (BCID) panel (bioMérieux, Salt Lake City, UT, USA, software version 3.2.4.0), a multiplex PCR-based platform that detects fungal and bacterial pathogens directly from flagged blood culture bottles. Matrix-assisted laser desorption/ionization time-of-flight mass spectrometry (MALDI-TOF MS; Bruker Daltonics GmbH & Co., FahrenheitstraBe 4, 28359 Bremen, Germany, version 5.2.360.235) was additionally available for species identification as required. Antifungal susceptibility testing was performed using the Sensititre system (Sensititre SWIN Software, Version 3.4-3.4.7.3, Thermo Fisher Scientific, UK) applied to all culture-positive isolates, with minimum inhibitory concentrations (MICs) reported as susceptible (S), intermediate (I), or resistant (R) per the laboratory reference ranges in use at KHCC. *C. krusei* was classified as intrinsically resistant to fluconazole per standard microbiological criteria.

### 2.7. Cluster Investigation and IPC Response

#### 2.7.1. Cluster Identification

The cluster was identified through prospective daily review of microbiology reports from 1 June 2025, following recognition of an above-background case frequency. All positive blood cultures were communicated to the infection control lead within 6 h of laboratory reporting. Twice-weekly interdisciplinary review meetings were established, comprising representatives from infectious diseases, pediatric oncology, clinical pharmacy, and nursing leadership.

#### 2.7.2. Individual Single-Room Isolation and Geographic Ward Sectioning

Given the profound immunocompromise of this patient population, in whom shared-room cohorting would constitute an unacceptable cross-infection risk, single-room isolation is standard institutional practice for all patients admitted to the unit at KHCC. Confirmed cases were housed within a geographically defined section comprising approximately half of the pediatric oncology ward, physically separated from the unaffected area by closed doors maintained throughout the cluster period.

#### 2.7.3. Contact Precautions

Contact precautions were implemented beyond standard hospital precautions and included mandatory donning of gloves and fluid-resistant gowns before any patient contact; dedicated equipment per patient (thermometers, blood pressure cuffs, and stethoscopes); twice-daily disinfection of high-touch surfaces with sodium hypochlorite 1000 ppm; and a dedicated nursing assignment at a minimum 1:2 ratio, with cross-assignment between isolation and general ward patients within a single shift prohibited.

#### 2.7.4. Hand Hygiene Promotion and Monitoring

The infection control team conducted regular hand hygiene monitoring rounds throughout the cluster period, comprising direct observation of hand hygiene practices and on-the-spot educational reinforcement for clinical and support staff. Observations focused on patient contact areas within the affected ward section, and findings were communicated verbally to ward leadership and nursing staff on the same day. No formal quantitative compliance data were prospectively recorded; hand hygiene monitoring is therefore described as a qualitative IPC reinforcement activity rather than a structured audit with reportable compliance rates.

#### 2.7.5. Visitor Restrictions

Visitor access was restricted to one designated primary caregiver per patient; sibling and extended-family visits were suspended for the duration of the active cluster. All caregivers received instruction on hand hygiene techniques and contact precautions at admission and at each nursing shift change.

#### 2.7.6. Antifungal Prophylaxis

Antifungal prophylaxis was introduced on 25 July 2025. In response to the active cluster, institutional eligibility criteria were expanded beyond standard practice to encompass all patients meeting any of the following high-risk criteria: (1) all AML patients (any line of therapy); (2) relapsed AML; (3) ALL during induction or re-induction with ANC ≤ 500 cells/µL; (4) ALL currently receiving or post re-intensification or FLAG-based induction/consolidation chemotherapy regimen; (5) relapsed ALL; and (6) high risk non-Hodgkin lymphomas. First-line prophylaxis was voriconazole administered per weight-based institutional dosing; micafungin 1 mg/kg/day intravenously (maximum 50 mg/day) was used when voriconazole was contraindicated. Prophylaxis was continued for the duration of severe neutropenia (ANC ≤ 500 cells/µL) and for a minimum of two weeks following neutrophil recovery. Following identification of azole-resistant *C. tropicalis* in two patients receiving voriconazole prophylaxis (Cases 4 and 6), micafungin was substituted as the preferred prophylaxis agent for all new initiations.

#### 2.7.7. Temporary Deferral of Myelosuppressive Chemotherapy

During the period of maximum concurrent case burden (operationally defined as ≥3 simultaneous active cases), initiation of new highly myelosuppressive chemotherapy cycles—defined as regimens expected to produce an absolute neutrophil count (ANC) nadir below 100 cells/µL for more than 7 consecutive days—was deferred pending joint oncology–infectious disease review. Approximately 20 patients due for intensive chemotherapy had initiation postponed, for approximately 3–4 weeks. All deferral decisions were individually documented, with family counseling recorded in the medical record.

#### 2.7.8. Environmental Investigation

A plumbing infrastructure failure with an associated water leak was identified in late August 2025 during the cluster investigation, based on visual and structural inspection of the affected ward area. No microbiological environmental sampling—including contact plates, air sampling, or water or surface cultures—was performed at any point during the investigation. Hospital water systems are a recognized source of nosocomial fungal contamination; biofilm accumulation in hospital plumbing can simultaneously harbor multiple *Candida* species, producing mixed-species clusters without clonal transmission [12]. The plumbing failure is accordingly described as a putative environmental source—temporally associated with the cluster and biologically plausible—but not microbiologically confirmed, as environmental cultures from affected surfaces were not obtained prior to structural repair. Remediation was completed in September 2025.

#### 2.7.9. CVC Care Audit

A dedicated audit of the CVC insertion technique, dressing care, and hub-handling practice was performed in the cluster by the infection control unit and infection control officers, using both direct observation and review of clinical and nursing documentation. This built on routine surveillance already in place, which was intensified during the outbreak from mid-July through early October 2025; monitoring continued past the decline in case counts to confirm sustained adherence. No breaches in aseptic technique were identified, and documentation showed consistent adherence to institutional insertion and dressing-change protocols.

### 2.8. Antifungal Therapy

All patients received therapy in accordance with IDSA 2016 candidemia management guidelines and the MASCC/ESCMID guidelines for fever and neutropenia management in children with cancer [14,15]. Empirical antifungal treatment was initiated within 24 h of clinical suspicion of candidemia, before species identification results were available. Micafungin was dosed at 2 mg/kg/day IV (maximum 100 mg/day); caspofungin at a loading dose of 70 mg/m^2^ followed by 50 mg/m^2^/day; and liposomal amphotericin B (L-AmB) at 3–5 mg/kg/day IV. Step-down or combination therapy was guided by species identification and susceptibility results, typically available by day 3–5 of treatment. CVC management followed current institutional and guideline-recommended protocols. Minimum treatment duration was 14 days following documented blood culture negativity and clinical resolution.

### 2.9. Statistical Analysis

Given the small cohort size (*n* = 7 confirmed cases) and the retrospective design without a concurrent control group, only descriptive statistics were performed. Categorical variables are presented as frequencies and percentages; continuous variables are presented as medians with ranges.

### 2.10. Ethics

This study was approved by the KHCC Institutional Review Board (approval number 26KHCC080; approved 6 April 2026). IRB approval included a waiver of individual informed consent.

## 3. Results

### 3.1. Culture-Confirmed Candidemia Cases

Seven pediatric inpatients with culture-confirmed candidemia were identified between 1 June and 30 September 2025 (Table 1). The cohort comprised six males and one female, with a median age of 12 years (range 1–17 years). All seven patients had a CVC in situ and had received broad-spectrum antibiotics within the preceding two weeks. Leukemia was the underlying diagnosis in 71% (5/7), comprising AML (n = 2) and ALL (n = 3). Four of seven patients (57%) required PICU admission.

### 3.2. Clinically Suspected IFI Cases

Five additional patients presenting during the same period met criteria for clinically suspected IFI based on compatible host risk factors and clinical or radiologic findings but had negative blood cultures throughout their clinical course (Cases 8–12). These patients are described for clinical completeness but are excluded from all candidemia-specific microbiological and outcome analyses (Table 2).

### 3.3. Microbiological Findings

*Candida tropicalis* was the predominant species among confirmed cases, isolated in four of seven patients (57%). *C. albicans*, *C. kefyr* and *C. krusei* were each identified in one patient (42% each). Azole resistance was identified in 42% of tested isolates (three cases out of seven cases in culture confirmed cases only): voriconazole resistance in two *C. tropicalis* isolates, and intrinsic fluconazole resistance in the one *C. krusei* isolate. Two of the three patients receiving voriconazole prophylaxis at candidemia onset harbored azole-resistant organisms (67%), representing prophylaxis breakthrough (Table 3). No echinocandin resistance was detected.

### 3.4. Cluster Timeline and Environmental Context

**Figure 1:**
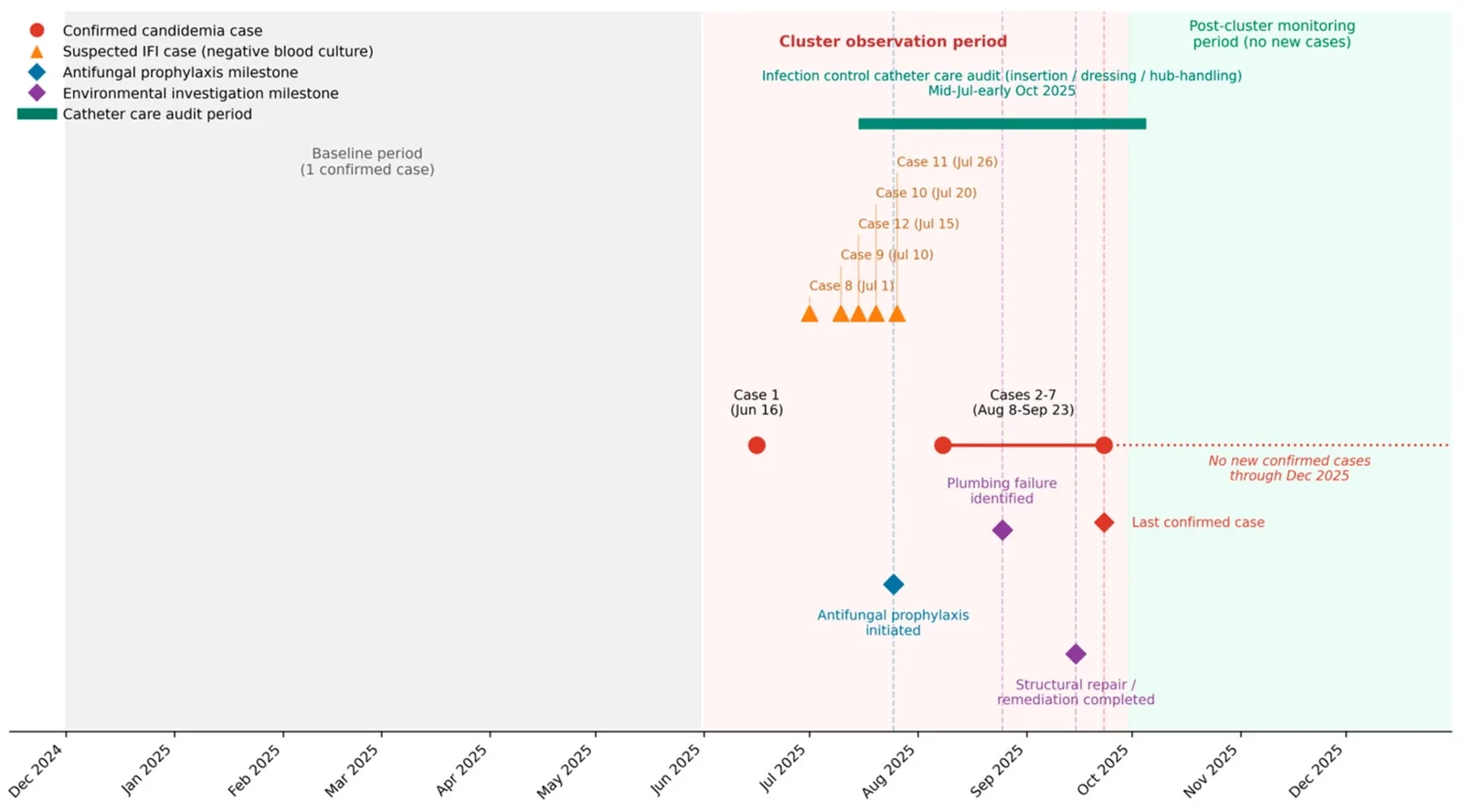
Timeline of the candidemia cluster, environmental investigation, catheter care audit, and IPC response at KHCC (December 2024–December 2025). Confirmed candidemia cases (red circles), clinically suspected IFI cases (orange triangles), and key environmental, prophylaxis, and audit milestones are shown relative to the cluster observation period (shaded) and the post-cluster monitoring period through December 2025, during which no new confirmed cases were identified.

The cluster spanned 1 June to 23 September 2025. One confirmed case was identified in June (first positive blood culture: 16 June); no new confirmed cases occurred throughout July; and the remaining six cases presented between 8 August and 23 September, with case frequency accelerating in August–September, coinciding with identification of the plumbing water leak in late August. Against a background rate of one confirmed case in the preceding six months, seven confirmed cases over a four-month period represent a seven-fold increase above background incidence (Figure 1).

Environmental investigation identified a plumbing infrastructure failure with an associated water leak in late August 2025. Structural repair was completed in September 2025. No new confirmed candidemia cases were identified after 23 September 2025, during the surveillance period. Environmental microbiological cultures from the affected surfaces were not obtained prior to remediation, which precludes microbiological confirmation of the water system as the source. No concurrent increase in bacteremia due to non-*Candida* pathogens was detected in the affected ward section during the same timeframe as the candidemia cluster.

### 3.5. Treatment and Tolerability

All seven confirmed candidemia patients received guideline-concordant antifungal therapy. L-AmB was the initial empirical agent in four cases (Cases 2, 3, 4, and 7); an echinocandin was used in three cases (Cases 1, 5, and 6). CVC removal was achieved in all patients within 48 h of candidemia diagnosis. Antifungal tolerability was acceptable; transient hypokalemia requiring supplementation occurred in three patients receiving amphotericin-based therapy, resolving without permanent renal impairment.

### 3.6. Outcomes

Six of seven confirmed candidemia patients completed antifungal therapy and were discharged. One patient (Case 1—Non-Langerhans Cell Histiocytosis, *C. kefyr*) died from septic shock attributable to candidemia, yielding a candidemia-attributable mortality of 14% (1/7). A second patient (Case 2—relapsed T-cell ALL) died from disease progression unrelated to the fungal infection; 30-day all-cause mortality among confirmed cases was 29% (2/7). Among the five clinically suspected IFI patients, two deaths were adjudicated to underlying malignancy.

## 4. Discussion

Between June and September 2025, 7 culture-confirmed candidemia cases occurred at KHCC over four months, against a background of one confirmed case in the preceding 6 months—a seven-fold incidence increase. The investigation identified a concurrent plumbing infrastructure failure as the putative environmental source. *C. tropicalis* was the predominant species (57%) with 42% azole resistance, consistent with regional epidemiological patterns. A structured IPC bundle was implemented, and no new confirmed cases were identified after 23 September 2025.

### 4.1. Case Definition Approach

The primary analysis was deliberately restricted to seven culture-confirmed candidemia cases, distinguishing these from five patients with clinically suspected IFI but negative blood cultures. This approach reflects established microbiological standards: blood culture positivity remains the accepted criterion for candidemia diagnosis, and clinical or radiologic findings alone cannot confirm *Candida* bloodstream infection in the absence of microbiological evidence [14]. Although this conservative stance may underestimate the true cluster magnitude—blood culture sensitivity is estimated at 63–83% for pure candidemia and as low as 21–71% when deep-seated disease predominates [16]—it ensures that all analyses and outcome data rest on verified microbiological evidence.

### 4.2. Cluster Epidemiology and Environmental Context

The seven-fold increase above the six-month background rate satisfies WHO criteria for a temporal cluster warranting investigation [13]. The heterogeneous species distribution—*C. tropicalis* (57%), *C. albicans* (14%), *C. kefyr* (14%) and *C. krusei* (14%)—is consistent with an environmental rather than a clonal source of acquisition, as hospital water system biofilms can simultaneously harbor multiple *Candida* species and produce mixed-species clusters without clonal transmission [12]. The identification of a plumbing infrastructure failure during the cluster investigation provides a temporally plausible putative source. However, the absence of environmental cultures prior to remediation precludes microbiological confirmation, and the cessation of new cases following structural repair represents a temporal association rather than proof of causality.

### 4.3. Catheter-Related Hypothesis

An alternative and biologically plausible explanation for this cluster is catheter-related acquisition, given that *Candida* species are common commensals of human skin and mucosal surfaces and all seven confirmed cases had a CVC in situ at the time of candidemia diagnosis; however, CVC presence was universal across the cohort and therefore does not, by itself, discriminate between a catheter-specific source and an environmental source. The species distribution observed—*C. tropicalis* (57%), *C. albicans* (14%), *C. kefyr* (14%) and *C. krusei* (14%)—is more consistent with a shared environmental reservoir capable of harboring multiple *Candida* species simultaneously than with a single catheter-handling breach, which would more typically be expected to produce a narrower, skin-flora-associated species profile or evidence of direct patient-to-patient (clonal) spread; molecular typing to formally test for clonality was not available at KHCC, which we acknowledge remains the central gap preventing definitive discrimination between the environmental and catheter-related hypotheses. This is further supported by a targeted audit of catheter care practice conducted during the cluster, which identified no breaches in aseptic technique, and by catheter-tip culture data: tips were sent for culture in three of the seven confirmed cases, all of which were negative, providing additional (though partial) evidence against a catheter-associated biofilm as the source in this subset, although catheter tip culture is not routinely performed at KHCC in the absence of specific clinical suspicion and was therefore not obtained for the remaining four cases—a partial dataset that we acknowledge in the Limitations as limiting our ability to fully exclude catheter-associated biofilms as a contributing source across the full cohort.

### 4.4. Species Distribution and Antifungal Resistance

The predominance of *C. tropicalis* and a 42% azole resistance rate (3 cases out of the 7 culture confirmed cases) are consistent with published surveillance data from LMIC pediatric oncology settings [6,7]. Azole resistance in *C. tropicalis* characteristically involves *ERG11* mutations conferring cross-resistance between fluconazole and voriconazole [8,9]. The observation that two of the three patients receiving voriconazole prophylaxis at candidemia onset harbored azole-resistant organisms—a prophylaxis-breakthrough rate of 67% (two cases had voriconazole resistance out of 3 cases who were on voriconazole prophylaxis from the total seven culture confirmed cases)—underscores the critical importance of local resistance surveillance in guiding prophylaxis selection. Furthermore, *C. tropicalis* demonstrates an increased propensity for disseminated disease in neutropenic hosts compared with other species, which has direct clinical implications for this predominantly leukemic cohort [17].

### 4.5. IPC Response

The IPC bundle comprised individual single-room isolation within a geographically sectioned ward, enhanced contact precautions, hand hygiene monitoring with direct educational reinforcement, expanded antifungal prophylaxis, and chemotherapy deferral during the period of maximum case burden. The last confirmed case was recorded on 23 September 2025, coinciding with completion of environmental remediation; no new confirmed cases were identified thereafter. Because these measures—individual-room isolation, contact precautions, hand hygiene monitoring, visitor restrictions, and environmental remediation—were implemented concurrently as a bundle rather than sequentially or individually, this retrospective, uncontrolled design cannot isolate the contribution of any single component to cluster resolution.

### 4.6. Clinical Outcomes

Candidemia-attributable mortality was 14% (1/7), and overall 30-day mortality was 29% (2/7), with the second death attributable to underlying malignancy rather than invasive fungal disease. These outcomes compare favorably with published mortality rates in analogous pediatric oncology populations: 36.5% at 28 days in Vietnamese pediatric leukemia cohorts [18], 36% at 30 days in an Indian pediatric cancer series [19], 20% in a Brazilian pediatric tertiary hospital cohort [20], and up to 55% in Turkish hematology–oncology series [7].

### 4.7. Limitations

This study has several important limitations. First, this study is limited by its small sample size (n = 7 confirmed cases) and single-center design, which restrict the generalizability of both the clinical findings and the effectiveness of the IPC bundle to other institutions. Second, the retrospective design meant that risk factor data were extracted from clinical documentation rather than prospective research records. Third, isolate storage and molecular typing—whether whole-genome sequencing (WGS) or multi-locus sequence typing (MLST)—were not available at KHCC, preventing determination of clonal versus polyclonal acquisition; this represents the most significant analytical gap in the cluster investigation. Fourth, environmental cultures from the water leak site were not collected before structural repair, preventing microbiological confirmation of the proposed environmental source. Fifth, blood culture sensitivity limitations—estimated at 63–83% for pure candidemia and 21–71% for deep-seated disease—mean the true cluster magnitude may be underestimated, particularly given the high rate of hepatosplenic involvement observed on imaging. Sixth, catheter tip cultures, obtained only for three of the seven confirmed cases as this is not routine practice at KHCC, were negative in all three; this partial dataset limits our ability to fully exclude catheter-associated biofilms as a contributing source across the full cohort. Finally, ward patient-day and catheter-day denominators were not available for either the baseline or cluster periods; incidence is therefore expressed as absolute case counts against a fixed comparison period rather than as a formal rate (e.g., per 1000 patient-days), which limits direct comparison with published incidence rates from other centers.

## 5. Conclusions

We describe a temporal candidemia cluster at a Jordanian pediatric oncology center, defined by a 7-fold increase in culture-confirmed candidemia above background incidence and coinciding with a novel environmental event. *C. tropicalis* accounted for 5 of 7 confirmed cases, and 42% azole resistance and two prophylaxis-breakthrough infections in patients receiving voriconazole prompted real-time substitution of micafungin, illustrating active antifungal stewardship in a resource-limited setting. A structured IPC bundle incorporating individual single-room isolation, geographic ward sectioning, enhanced contact precautions, and expanded antifungal prophylaxis was fully implemented with defined operational criteria. Candidemia-attributable mortality among confirmed cases was 14%. These data contribute to the limited published literature on candidemia clusters in Middle Eastern pediatric oncology centers and demonstrate that structured IPC responses are operationally achievable without advanced microbiological or genomic infrastructure.

## Figures and Tables

**Table 1:** Clinical Characteristics, Microbiological Findings, Antifungal Management, and Outcomes of Culture-Confirmed Candidemia Cases (n = 7).

Case	Age (yr)	Sex	Diagnosis	CVC	Disease Status	Candida Species	Antifungal Therapy	Prophylaxis at Onset	Outcome
1	1	F	Non-Langerhans Cell Histiocytosis	Yes	Non remission	*C. kefyr*	Micafungin	None	Died *
2	6	M	Relapsed T-cell ALL	Yes	Non remission	*C. tropicalis* [R]	L-AmB → Caspofungin	None	Died **
3	17	M	T-cell ALL	Yes	remission	*C. krusei* [intrinsic R]	L-AmB + Micafungin → Fluconazole step-down	None	Alive
4	15	M	AML	Yes	Non remission	*C. tropicalis* [R]	L-AmB 3	Voriconazole	Alive
5	12	M	Relapsed B ALL	Yes	Non remission	*C. tropicalis*	Caspofungin	Voriconazole	Alive
6	14	M	AML	Yes	Non remission	*C. tropicalis* [R]	Caspofungin	Voriconazole	Alive
7	13	M	Low-grade glioma	Yes	stable	*C. albicans*	L-AmB → Fluconazole step-down	None	Alive

[R] = azole-resistant isolate; [intrinsic R] = intrinsically fluconazole-resistant. * Death attributable to candidemia. ** Death attributable to underlying malignancy/disease progression. ALL = acute lymphoblastic leukemia; AML = acute myeloid leukemia; CVC = central venous catheter; d = days; L-AmB = liposomal amphotericin B; yr = years.

**Table 2:** Clinical Characteristics and Outcomes of Clinically Suspected Invasive Fungal Infection (IFI) Cases (Secondary Descriptive Group, n = 5), Excluded from Candidemia-Specific Analyses.

Case	Age (yr)	Sex	Diagnosis	CVC	Disease Status	Candida Species	Antifungal Therapy	Prophylaxis at Onset	Outcome
8	3	M	B-cell ALL	Yes	remission	Not isolated (suspected IFI)	L-AmB + Micafungin → Voriconazole	None	Alive
9	12	M	B-cell ALL	Yes	remission	Not isolated (suspected IFI)	L-AmB + Micafungin → Voriconazole	Micafungin	Alive
10	2	M	AML	Yes	remission	Not isolated (suspected IFI)	L-AmB + Micafungin → Voriconazole	None	Alive
11	17	M	Low-grade glioma	Yes	stable	Not isolated (suspected IFI)	L-AmB → Voriconazole	None	Died **
12	10	M	AML	Yes	Non remission	Not isolated (suspected IFI)	L-AmB → Voriconazole	None	Died **

** Death attributable to underlying malignancy/disease progression. ALL = acute lymphoblastic leukemia; AML = acute myeloid leukemia; CVC = central venous catheter; d = days; IFI = invasive fungal infection; L-AmB = liposomal amphotericin B; yr = years.

**Table 3:** Antifungal Resistance Patterns—Culture-Confirmed Cases Only.

Case	Species	Prophylaxis at Onset	Resistance Pattern	MIC Voriconazole (mg/L)	MIC Fluconazole (mg/L)	Outcome
2	*C. tropicalis*	None	Voriconazole-resistant	≥1	Susceptible	Died
4	*C. tropicalis*	Voriconazole	Voriconazole + fluconazole-resistant	≥1	≥8	Alive
6	*C. tropicalis*	Voriconazole	Voriconazole + fluconazole-resistant	≥1	≥8	Alive

Cases 4 and 6 were receiving voriconazole prophylaxis at candidemia onset; both harbored azole-resistant isolates, representing prophylaxis-breakthrough infections. MIC = minimum inhibitory concentration; results reported as susceptible, intermediate, or resistant per KHCC laboratory reference ranges.

## Data Availability

De-identified data are available upon reasonable request from the corresponding author.
